# Icariin improves metabolic response to exercise by promoting TFEB-dependent mitochondrial clearance and metabolic reprogramming in C57BL/6 mice and C2C12 myotubes

**DOI:** 10.3389/fnut.2026.1754850

**Published:** 2026-04-13

**Authors:** Zhengyuan Liu, He Hu, Liang Zheng

**Affiliations:** 1 College of Physical Education, Keimyung University, Daegu, Republic of Korea; 2 College of Sports and Health, Guilin Institute of Information Technology, Guilin, Guangxi, China

**Keywords:** anti-fatigue, icariin, lactate, mitophagy, TFEB

## Abstract

**Background:**

Fatigue during intensive exercise is closely associated with metabolic inefficiency and lactate accumulation. While Icariin, a natural flavonoid, has demonstrated potential in enhancing exercise performance, the precise cellular mechanisms governing its anti-fatigue effects remain incompletely elucidated.

**Methods:**

We employed an integrated approach combining *in vivo* exercise models in C57BL/6 mice with *in vitro* C2C12 myotube systems. Mice received Icariin supplementation (50 or 100 mg/kg) for 4 weeks before comprehensive physiological assessments. Cellular studies utilized caffeine stimulation, transcriptomic profiling, and metabolic analyses. Molecular mechanisms were investigated through western blotting, immunofluorescence, and genetic knockdown approaches.

**Results:**

Icariin supplementation dose-dependently enhanced exercise performance, evidenced by increased maximal oxygen consumption (VO_2_max) and prolonged exhaustive running time. This improvement was accompanied by reduced blood lactate accumulation, skeletal muscle hypertrophy, and a shift toward oxidative fiber types. In C2C12 myotubes, Icariin directly attenuated lactate production by suppressing LDH activity and reprogramming cellular metabolism toward oxidative phosphorylation. Transcriptomic analysis revealed significant enrichment of mitophagy pathways, which was validated by enhanced mitophagic flux and improved mitochondrial membrane potential. Mechanistically, we identified TFEB as the key transcriptional regulator mediating Icariin's effects, evidenced by its dephosphorylation, nuclear translocation, and transactivation of mitophagic genes. Crucially, TFEB knockdown completely abolished Icariin-induced mitophagy, metabolic improvements, and lactate reduction.

**Conclusion:**

Our findings establish a comprehensive mechanistic pathway wherein Icariin activates TFEB to drive mitophagic clearance of dysfunctional mitochondria, thereby optimizing mitochondrial function and shifting energy metabolism toward oxidative phosphorylation. This TFEB-mitophagy axis represents the core mechanism through which Icariin enhances exercise performance and metabolic efficiency, providing novel insights into its anti-fatigue properties and potential therapeutic applications.

## 1 Introduction

Skeletal muscle fatigue during intensive exercise represents a complex physiological state characterized by a reversible decline in force-generating capacity and exercise intolerance. This phenomenon is intimately associated with metabolic perturbations, among which the accumulation of lactic acid due to an overreliance on anaerobic glycolysis is a well-established contributor (1, 2). The resulting intracellular acidification impairs calcium handling and contractile function, while the inefficient ATP yield from glycolysis fails to meet the energetic demands of sustained muscle work (3, 4). While adaptive processes such as enhanced mitochondrial biogenesis and a shift toward oxidative muscle fibers can ameliorate metabolic efficiency and fatigue resistance (5, 6), the precise molecular regulators that can be therapeutically targeted to orchestrate these adaptations remain incompletely defined.

Icariin, a prenylated flavonoid glycoside derived from the *Epimedium* genus (Berberidaceae), has attracted increasing scientific interest for its potential ergogenic and anti-fatigue properties. Previous pharmacological studies have demonstrated that Icariin and its metabolites exhibit favorable pharmacokinetic profiles with good tissue distribution, particularly in bone and muscle tissues (7, 8). Animal studies have consistently reported that Icariin supplementation can extend exhaustive swimming time and improve load-bearing capacity in rodents (9, 10). Proposed mechanisms from these initial investigations include the attenuation of exercise-induced oxidative stress through enhancement of endogenous antioxidant systems (11), modulation of neuroendocrine responses (12), and improvement of skeletal muscle energy metabolism through AMPK pathway activation (13). However, a significant gap persists in our understanding. The direct cellular targets within skeletal muscle and the overarching transcriptional programs through which Icariin systematically reprograms muscle metabolism to enhance fatigue resistance are still largely unknown.

The maintenance of mitochondrial quality through selective autophagic clearance of damaged organelles, known as mitophagy, has emerged as a crucial determinant of skeletal muscle metabolic health and exercise adaptation (14, 15). During prolonged exercise, mitochondria are subjected to increased reactive oxygen species production and calcium overload, leading to mitochondrial membrane depolarization and dysfunction (16). The efficient removal of these damaged mitochondria through mitophagy is therefore essential for preserving the functional integrity of the mitochondrial network and sustaining energy production during repeated contractions (17). Impairments in mitophagic flux have been linked to accelerated muscle fatigue and metabolic inefficiency in aging and disease states (18, 19). Given that previous studies have demonstrated Icariin's ability to activate AMPK and enhance antioxidant defense—pathways intimately linked to autophagy regulation—it is plausible that Icariin may exert its anti-fatigue effects, at least in part, through modulation of mitochondrial quality control. Indeed, several bioactive compounds with structural or functional similarities to Icariin, such as resveratrol and flavonoids, have been shown to enhance mitochondrial quality through activation of autophagic pathways (20). Yet, whether Icariin directly influences this critical quality control process remains entirely unexplored.

The transcription factor EB (TFEB) has emerged as a master regulator of lysosomal biogenesis and autophagy, including mitophagy (21). Under basal conditions, TFEB resides in the cytoplasm in a phosphorylated state, but upon activation undergoes dephosphorylation and nuclear translocation, where it binds to Coordinated Lysosomal Expression and Regulation (CLEAR) elements in target gene promoters (22, 23). By transactivating a coherent set of genes involved in autophagosome formation, lysosomal function, and mitochondrial clearance, TFEB orchestrates a comprehensive cellular clearance and renewal program (24). Recent evidence has implicated TFEB activation in the beneficial effects of certain phytochemicals, such as resveratrol and curcumin, on mitochondrial function (25, 26). Furthermore, exercise itself has been shown to activate TFEB in skeletal muscle, suggesting its fundamental role in activity-induced adaptations (27).

Despite these advances, several critical questions remain unanswered: At the cellular level, does Icariin directly enhance mitochondrial quality through activation of mitophagy in skeletal muscle cells, and is TFEB involved in mediating these effects in C2C12 myotubes? At the organismal level, does Icariin administration activate TFEB and promote mitophagy in skeletal muscle of C57BL/6 mice, and most importantly, does this TFEB-mitophagy axis represent the fundamental mechanism through which Icariin improves exercise performance *in vivo*? To address these questions, we employed an integrated approach combining *in vivo* physiological assessments in a murine model with in-depth molecular and cellular investigations. This study provides the **first** comprehensive evidence that Icariin enhances exercise performance through TFEB-mediated activation of mitophagy, revealing a novel mechanistic pathway that connects natural product supplementation to improved skeletal muscle metabolic efficiency.

## 2 Materials and methods

### 2.1 Animals and experimental design

Eight-week-old male C57BL/6J mice (*n* = 24) with a mean body weight of 21 ± 1 g were obtained from Cyagen Laboratory Animal Technology Co., Ltd (Suzhou, China). The sample size was determined based on previous studies investigating exercise performance in mice, using power analysis (α = 0.05, power = 0.8) to detect a 20% difference in exhaustive running time with an estimated effect size of 1.5. Animals were randomly allocated to three experimental groups using a computer-generated randomization sequence. All mice were group-housed (4 mice per cage) in standard IVC cages (dimensions: 365 × 207 × 140 mm) with corn cob bedding under specific pathogen-free (SPF) conditions. The housing environment was maintained at 22 ± 2 °C with 50 ± 5% relative humidity and a 12/12 h light/dark cycle (lights on at 07:00). Animals had *ad libitum* access to standard laboratory chow and autoclaved water. Environmental enrichment included nesting material and a plastic shelter. All cages were changed twice weekly.

After 1 week of acclimatization, mice were randomly divided into three experimental groups (*n* = 8 per group):

(1) Control group: administered vehicle (phosphate-buffered saline, PBS) daily

(2) Low-dose Icariin group: administered 50 mg/kg/day Icariin

(3) High-dose Icariin group: administered 100 mg/kg/day Icariin (28, 29)

Icariin (purity ≥98%, HPLC; MedChemExpress, HY-N0014) was dissolved in PBS and administered by oral gavage in a volume of 10 mL/kg body weight daily for 4 consecutive weeks. The vehicle control group received equivalent volumes of PBS. Body weight and general health status were monitored and recorded weekly throughout the experimental period.

All animal experiments were conducted in accordance with the National Institutes of Health Guide for the Care and Use of Laboratory Animals and were approved by the Animal Ethics Committee of Keimyung University (Approval No. 2024-KMU1703). All procedures were designed to minimize animal suffering and used the minimum number of animals necessary to obtain scientifically valid results.

### 2.2 *In vivo* exercise performance tests

All exercise tests were conducted during the dark phase of the light cycle. After the 4-week supplementation period, exercise performance was evaluated as follows:

#### 2.2.1 Maximal oxygen consumption (VO_2_max)

Mice were subjected to an incremental running protocol on a motorized treadmill. The protocol started at a speed of 10 m/min with a 5° incline. The speed was increased by 3 m/min every 3 min until exhaustion. Exhaustion was defined as the inability of the mouse to return to the treadmill despite manual encouragement for 10 consecutive seconds. VO_2_ and VCO_2_ were measured in real-time using an open-circuit calorimetry system. VO_2_max was determined as the highest average O_2_ consumption over a 30 s period.

#### 2.2.2 Endurance running test

48 h after the VO_2_max test, mice ran at a constant speed corresponding to 85% of their individual pre-determined VO_2_max. The time to exhaustion was recorded.

#### 2.2.3 Blood lactate measurement

A separate cohort of mice was used. Blood samples (5 μL) were collected from the tail tip at rest and immediately after a 15 min run at a fixed speed of 15 m/min. Blood lactate concentration was measured using a portable lactate analyzer (Lactate Scout 4, SensLab GmbH).

### 2.3 Sample collection and histological analysis

Mice were euthanized 48 h after the last exercise test. The gastrocnemius and soleus muscles from both hind limbs were rapidly dissected, weighed for wet weight measurement, and processed for subsequent analysis. For histology, a portion of the gastrocnemius muscle was fixed in 4% paraformaldehyde (PFA) in PBS for 24 h, dehydrated, embedded in paraffin, and sectioned into 5 μm thick cross-sections. Sections were stained with Hematoxylin and Eosin (H&E) following standard protocols. Images were captured using a microscope equipped with a digital camera. The cross-sectional area (CSA) of at least 200 myofibers per mouse was analyzed using ImageJ software.

### 2.4 Cell culture and treatments

The mouse C2C12 myoblast cell line was cultured in growth medium consisting of Dulbecco's Modified Eagle Medium (DMEM) supplemented with 10% fetal bovine se-rum (FBS) and 1% penicillin/streptomycin at 37 °C in a humidified atmosphere of 5% CO_2_. To induce differentiation into myotubes, when cells reached 90–100% confluence, the growth medium was replaced with differentiation medium (DMEM containing 2% horse serum). The medium was changed every 48 h, and myotubes were used for experiments after 4–5 days of differentiation.

For drug treatments, differentiated C2C12 myotubes were pretreated with various concentrations of Icariin (5–20 μM) (30) or vehicle control for 24 h. To model exercise-like stress *in vitro*, cells were stimulated with caffeine (5 mM) for 4 h in serum-free, low-glucose DMEM.

### 2.5 Biochemical assays

#### 2.5.1 Lactate measurement

The lactate concentration in the cell culture medium was measured using a commercial Lactate Assay Kit (AB65331, Abcam) according to the manufacturer's instructions.

#### 2.5.2 LDH activity assay

Intracellular LDH activity was measured from cell lysates using a Lactate Dehydrogenase Activity Assay Kit (EEA013, ThermoFisher).

#### 2.5.3 Cell viability (MTT) assay

Cell viability was assessed using the MTT assay. Briefly, cells were incubated with 0.5 mg/mL MTT reagent for 4 h at 37 °C. The formed formazan crystals were dissolved in DMSO, and the absorbance was measured at 570 nm with a reference wavelength of 630 nm using a microplate reader.

### 2.6 RNA extraction, reverse transcription, and quantitative real-time PCR (qRT-PCR)

Total RNA was extracted from cells or frozen muscle tissues using TRIzol Reagent according to the manufacturer's protocol. RNA concentration and purity were determined using a NanoDrop spectrophotometer. 1 μg of total RNA was reverse-transcribed into cDNA using a PrimeScript RT reagent Kit with gDNA Eraser. qRT-PCR was performed on a QuantStudio 3 Real-Time PCR System using SYBR Green Premix Pro Taq HS qPCR Kit. The relative mRNA expression levels were calculated using the $2^{-\Delta \Delta {\text{C}}_{T}}$ method and normalized to the expression of the housekeeping gene Gapdh. The primer sequences used in this study are listed in Supplementary Table 1.

### 2.7 Western blot analysis

Tissues or cells were lysed in RIPA lysis buffer supplemented with 1% protease and phosphatase inhibitor cocktail. Protein concentrations were determined using a BCA Protein Assay Kit. Equal amounts of protein (20 μg) were separated by SDS-PAGE on 10% gels and transferred onto PVDF membranes. The membranes were blocked with 5% non-fat milk in TBST for 1 h at room temperature and then incubated with primary antibodies at 4 °C overnight. The primary antibodies used were: anti-MyHC I (MA5-48095, ThermoFisher), anti-MyHC IIb (#3403, CST), anti-TFEB (#13372-1-AP, Proteintech), anti-phospho-TFEB (Ser211) (#37681, CST), and anti-GADPH (#60004-1, Proteintech). After washing, membranes were incubated with appropriate HRP-conjugated secondary antibodies for 1 h at room temperature. Protein bands were visualized using an enhanced chemiluminescence (ECL) substrate and imaged with a chemiluminescence imaging system. Densitometric analysis was performed using ImageJ software.

### 2.8 Transcriptomic sequencing and bioinformatic analysis

Total RNA was extracted from C2C12 myotubes treated with 20 μM Icariin or vehicle for 24 h (*n* = 3 per group). RNA integrity was verified using an Agilent 2100 Bioanalyzer. Library construction and sequencing were performed by Lianchuan Biotechnology on an Illumina NovaSeq 6000 platform to generate 150 bp paired-end reads. Raw reads were processed and aligned to the mouse reference genome (GRCm39). Differential expression analysis was performed using the DESeq2 R package, with genes having |log_2_(Fold Change)| > 1 and an adjusted *p*-value < 0.05 considered differentially expressed genes (DEGs). Gene Ontology (GO) enrichment analysis and Gene Set Enrichment Analysis (GSEA) were conducted. Transcription factor prediction was performed using the TTRUST database.

### 2.9 Mitochondrial function assessment

#### 2.9.1 High-resolution respirometry

Mitochondrial respiratory function in skeletal muscle homogenates was assessed using the Oroboros O2k-FluoRespirometer. State 3 respiration was measured in MiR05 respiration buffer following the addition of ADP in the presence of glutamate and malate.

#### 2.9.2 Seahorse XF cell mito stress test

The oxygen consumption rate (OCR) and extracellular acidification rate (ECAR) of C2C12 myotubes were measured using a Seahorse XFe96 Analyzer. The assay was performed according to the standard protocol by sequentially injecting oligomycin, FCCP, and rotenone/antimycin A.

### 2.10 Mitophagy and mitochondrial membrane potential assays

#### 2.10.1 Mito-Keima assay

C2C12 myotubes were transfected with the ptfLC3-mito-Keima plasmid using Lipofectamine 3000. After treatment, cells were analyzed by flow cytometry. Mitophagic flux was quantified by calculating the ratio of the acidified (excited at 561 nm) to non-acidified (excited at 488 nm) Keima signal.

### 2.11 Immunofluorescence staining

C2C12 myotubes grown on glass coverslips were fixed with 4% PFA, permeabilized with 0.1% Triton X-100, and blocked with 5% BSA. Cells were then incubated with anti-TFEB primary antibody overnight at 4 °C, followed by incubation with an Alexa Fluor 488-conjugated secondary antibody. Nuclei were counterstained with DAPI. Images were captured using a confocal microscope.

### 2.12 RNA interference

C2C12 myotubes were transfected with siRNA targeting mouse Tfeb or a non-targeting control siRNA (si-Scramble) using Lipofectamine RNAiMAX Transfection Reagent according to the manufacturer's instructions. Cells were harvested 48–72 h post-transfection for subsequent experiments. Knockdown efficiency was verified by western blotting.

### 2.13 *Ex vivo* muscle contractility

The extensor digitorum longus (EDL) muscle was carefully dissected and mounted in an organ bath containing Krebs-Ringer solution bubbled with 95% O_2_ and 5% CO_2_ at 25 °C. The muscle was stimulated with electrical pulses (100 Hz, 500 ms duration, 2 s interval) via parallel electrodes. Isometric contractile force was measured using a force transducer and recorded using LabChart software.

### 2.14 Statistical analysis

All data are presented as mean ± standard deviation (SD). Statistical analyses were performed using GraphPad Prism software (version 9.0). Differences between two groups were analyzed by Student's *t*-test. For multiple group comparisons, one-way analysis of variance (ANOVA) followed by Tukey's *post hoc* test was used. A *p*-value of less than 0.05 was considered statistically significant.

## 3 Results

### 3.1 Icariin supplementation enhances exercise performance in mice by improving skeletal muscle function and mitochondrial energetics

**Figure 1:**
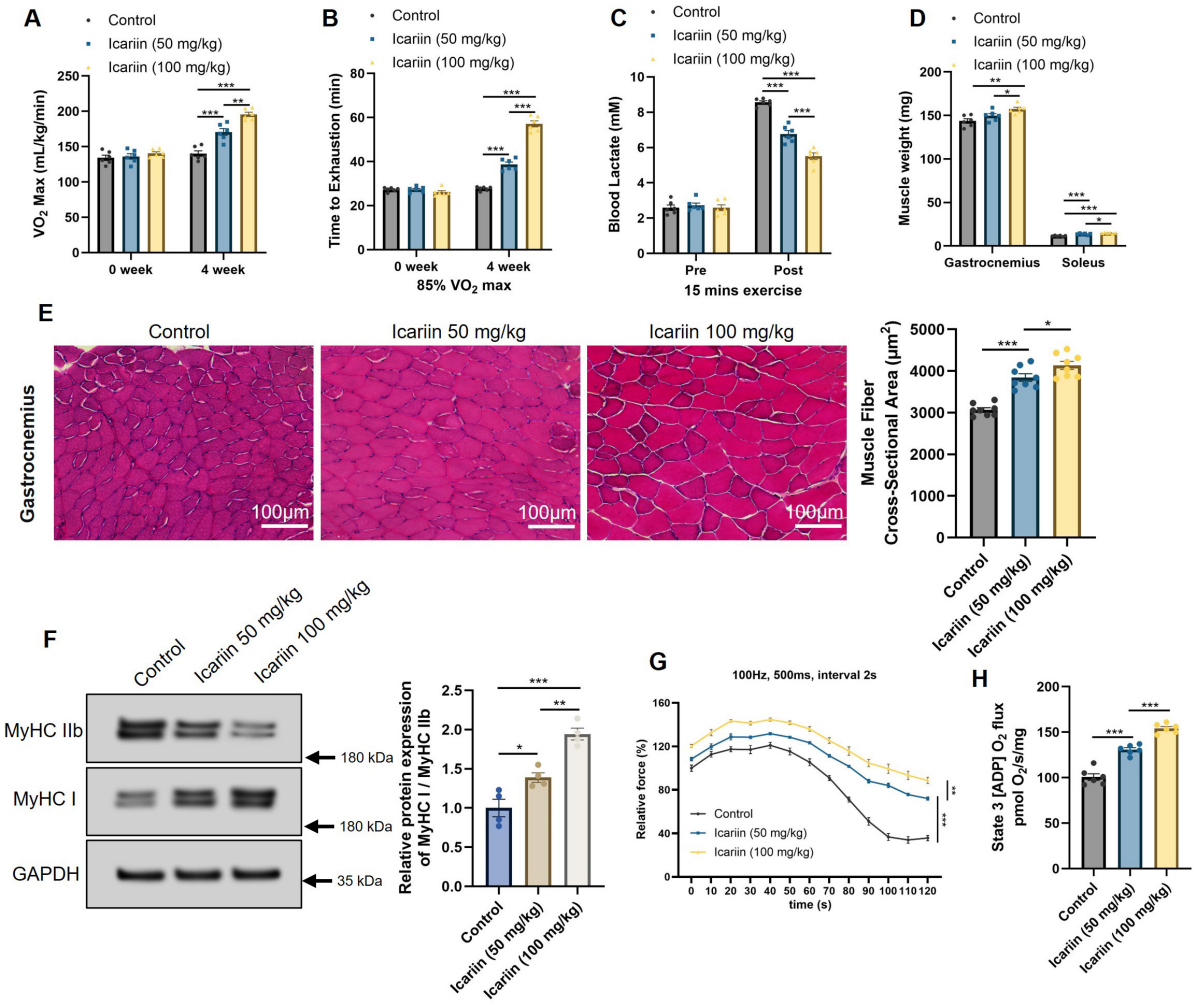
Effects of Icariin supplementation on exercise performance and skeletal muscle adaptation in mice. To assess exercise performance, mice underwent metabolic treadmill testing to determine **(A)** maximal oxygen consumption and **(B)** endurance capacity, measured as time to exhaustion at 85% of individual VO_2_max. **(C)** Blood lactate levels were evaluated before and after a 15 min run. **(D)** Post-sacrifice, skeletal muscle adaptation was analyzed by measuring the wet weights of the gastrocnemius and soleus muscles. **(E)** Cross-sectional area (CSA) of myofibers from gastrocnemius muscle was quantified from H&E-stained sections. **(F)** The protein ratio of Myosin Heavy Chain (MyHC) I to MyHC IIb in gastrocnemius muscle was determined by west-ern blot. **(G)** Contractileforce of isolated extensor digitorum longus (EDL) muscles was measured *ex vivo* in response to electrical stimulation. **(H)** Mitochondrial respiratory function in skeletal muscle homogenates was assessed by high-resolution respirometry, showing O_2_ consumption rates under State 3 (ADP-stimulated) conditions. All data are presented as mean ± SEM. Sample size: *n* = 8 mice per group. Statistical comparisons between two groups were performed using unpaired two-tailed Student's *t*-test. Significance thresholds: *p < 0.05, **p < 0.01, ***p < 0.001 vs. control group.

To evaluate the anti-fatigue effect of Icariin, mice were subjected to a 4 week supplementation followed by comprehensive exercise tests. After the treatment period, both the 50 mg/kg and 100 mg/kg Icariin groups exhibited a significant increase in maximal oxygen consumption (VO_2_max) compared to the control group, with the higher dose producing a more pronounced effect (Figure 1A). Subsequently, we assessed endurance performance by measuring the time to exhaustion at a running speed corresponding to 85% of their individual VO_2_max. Consistent with the VO_2_max data, Icariin treatment markedly prolonged the exhaustive running time in a dose-dependent manner, with the 100 mg/kg group demonstrating superior performance (Figure 1B). Given the critical role of lactate in fatigue, we measured blood lactate levels pre- and post-exercise. Icariin supplementation did not affect resting lactate levels but significantly blunted the sharp increase in blood lactate following a 15 min run (Figure 1C), indicating an enhanced metabolic efficiency and reduced reliance on anaerobic glycolysis during exercise. Furthermore, gross morphological analysis revealed that Icariin administration significantly increased the wet weight of both the gastrocnemius (a mixed fiber type muscle) and the soleus (a slow-twitch dominant muscle), again in a dose-dependent fashion (Figure 1D). Histological examination of the gastrocnemius via H&E staining confirmed a substantial increase in the cross-sectional area (CSA) of myofibers in Icariin-treated mice (Figure 1E), demonstrating that the improved performance was associated with clear skeletal muscle hypertrophy. To delve into the molecular basis for the enhanced endurance, we analyzed the composition of myosin heavy chain (MyHC) isoforms. Western blot analysis showed that Icariin treatment significantly increased the ratio of MyHC I (a marker for slow-twitch, fatigue-resistant fibers) to MyHC IIb (a marker for fast-twitch, glycolytic fibers) (Figure 1F). This shift toward a more oxidative phenotype is consistent with the observed improvement in endurance. We further evaluated the functional outcome of this remodeling using *ex vivo* muscle contractility assays. Upon electrical stimulation, isolated muscles from Icariin-treated mice exhibited a significant, dose-dependent increase in contractile force (Figure 1G), directly linking the structural and molecular changes to superior muscle performance. Finally, to identify the upstream mechanism driving these adaptations, we assessed mitochondrial function. High-resolution respirometry on muscle homogenates revealed that Icariin significantly boosted State 3 respiration (ADP-stimulated respiration), reflected by an increased O_2_ flux (Figure 1H). This demonstrates a fundamental enhancement in mitochondrial oxidative phosphorylation capacity, which provides the necessary energetic foundation for the observed fiber-type switching, reduced lactate production, and improved endurance.

### 3.2 Icariin directly attenuates lactate production in C2C12 myotubes by reprogram-ming cellular energy metabolism

**Figure 2:**
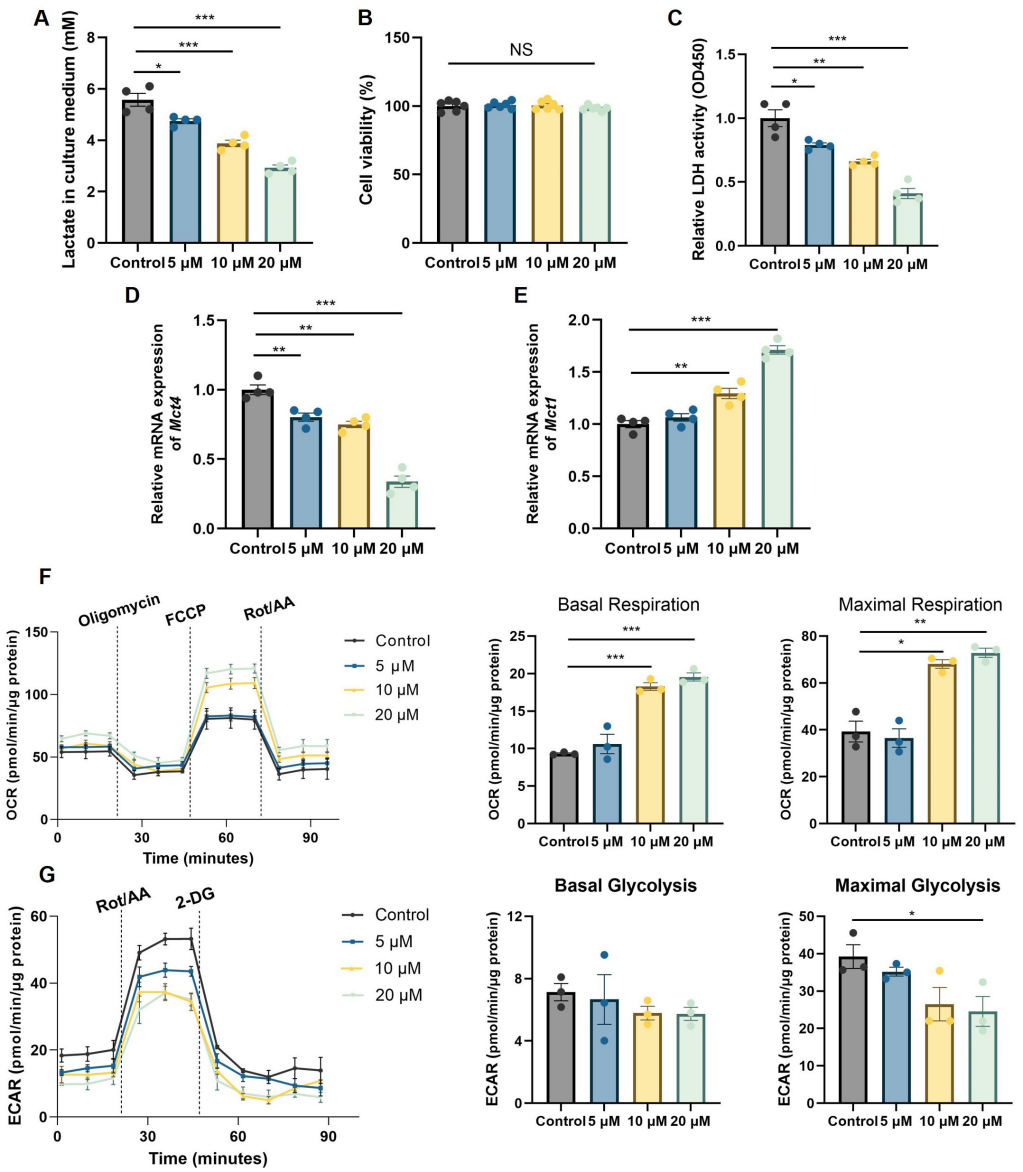
Icariin reprograms energy metabolism and attenuates lactate production in C2C12 myotubes. **(A)** Lactate concentration in the culture medium of caffeine-stimulated C2C12 myotubes treated with Icariin. **(B)** Cell viability of C2C12 myotubes treated with Icariin, assessed by MTT assay. **(C)** Lactate dehydrogenase (LDH) enzyme activity in cell lysates from Icariin-treated myotubes. **(D, E)** mRNA expression levels of the lactate transporters MCT4 **(D)** and MCT1 **(E)**, measured by qRT-PCR. **(F, G)** Cellular metabolic profiles assessed using the Seahorse XF Analyzer, showing **(F)** mitochondrial oxygen consumption rate (OCR) and **(G)** extracellular acidification rate (ECAR). All data are presented as mean ± SEM. Sample size: *n* = 3–4 independent experiments (with 3–6 technical replicates each), as indicated by the individual data points in the figures. Statistical comparisons between two groups were performed using unpaired two-tailed Student's *t*-test; for multiple groups, one-way ANOVA followed by Tukey's *post hoc* test was used. Significance thresholds: *p < 0.05, **p < 0.01, ***p < 0.001 vs. control group (unless otherwise indicated by brackets).

To elucidate the direct cellular mechanisms underlying the reduced lactate levels observed *in vivo*, we employed a caffeine-stimulated C2C12 myotube model. Consistent with our animal findings, Icariin treatment dose-dependently reduced caffeine-induced lactate secretion into the culture medium (Figure 2A). This effect was not attributable to cytotoxic activity, as MTT assays confirmed that the tested concentrations of Icariin did not compromise cell viability (Figure 2B).

We subsequently investigated the mechanistic basis for the decreased lactate output. Icariin treatment significantly suppressed the activity of lactate dehydrogenase (LDH), the key enzyme responsible for converting pyruvate to lactate (Figure 2C), thereby directly limiting the terminal step of anaerobic glycolysis. Concurrently, analysis of lactate transporter expression revealed that Icariin upregulated the mRNA expression of MCT1, which facilitates lactate uptake, while downregulating MCT4, which mediates lactate export (Figures 2D, E). This coordinated shift in transporter profile suggests a promotion of lactate clearance and utilization, rather than its extrusion.

Given that the balance between mitochondrial oxidation and glycolytic flux is a primary determinant of lactate accumulation, we profiled the cellular energy metabolism using the Seahorse XF Analyzer. Icariin treatment dose-dependently enhanced mitochondrial oxidative phosphorylation while concurrently suppressing glycolytic capacity (Figures 2F, G). This metabolic reprogramming toward a more oxidative phenotype provides a fundamental explanation for the observed reduction in LDH activity and the consequent decrease in lactate production. Collectively, these *in vitro* data demonstrate that Icariin directly targets skeletal muscle cells to orchestrate a metabolic shift from glycolysis to mitochondrial respiration, which underpins its anti-fatigue effect by limiting lactic acid accumulation.

### 3.3 3 Transcriptomic profiling reveals that icariin induces a mitophagic program to ameliorate metabolic efficiency

**Figure 3:**
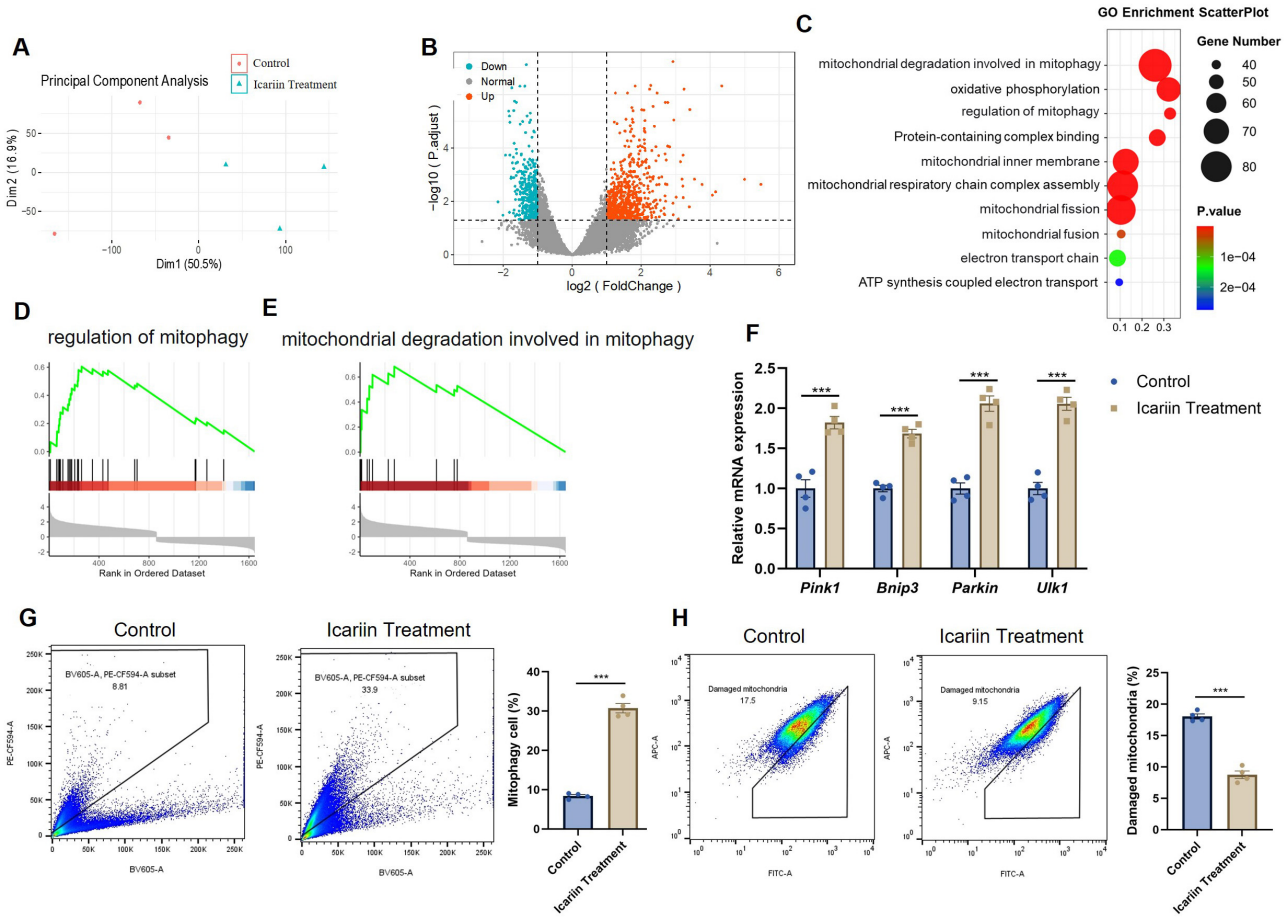
Transcriptomic analysis reveals Icariin-induced activation of mitophagy. **(A)** Principal component analysis (PCA) plot of RNA-sequencing data from control and Icariin-treated C2C12 myotubes. **(B)** Volcano plot showing differentially expressed genes (DEGs) between control and Icariin-treated groups. **(C)** Gene Ontology (GO) enrichment analysis of biological processes for the upregulated DEGs. **(D, E)** Gene Set Enrichment Analysis (GSEA) plots for the gene sets “regulation of mitophagy” **(D)** and “mitochondrial degradation involved in mitophagy” **(E)**. **(F)** qRT-PCR analysis of mRNA expression for key mitophagy-related genes (Pink1, Parkin, Bnip3, Ulk1). **(G)** Mitophagic flux was quantified by flow cytometry in C2C12 myotubes expressing the Mito-Keima probe. **(H)** The proportion of damaged mitochondria was assessed by flow cytometry using MitoTracker Red CMXRos staining. All data are presented as mean ± SEM. Sample size: *n* = 3 independent experiments with 3 technical replicates each, as indicated by the individual data points in the figures. Statistical comparisons between two groups were performed using unpaired two-tailed Student's *t*-test; for multiple groups, one-way ANOVA followed by Tukey's *post hoc* test was used. Significance thresholds: *p < 0.05, **p < 0.01, ***p < 0.001 vs. control group (unless otherwise indicated by brackets).

To gain an unbiased insight into the global mechanism by which Icariin reprograms cellular metabolism, we performed RNA-sequencing on C2C12 myotubes treated with or without 20 μM Icariin. Principal component analysis (PCA) demonstrated a clear segregation between the transcriptomic profiles of the control and Icariin-treated groups (Figure 3A). Using a threshold of |log_2_(Fold Change) | > 1 and an adjusted *p*-value < 0.05, we identified 1919 differentially expressed genes (DEGs), comprising 1134 upregulated and 785 downregulated genes (Figure 3B).

Gene Ontology (GO) enrichment analysis of the upregulated DEGs revealed a significant overrepresentation of terms related to mitochondrial quality control and function, including “regulation of mitophagy,” “mitochondrial degradation involved in mitophagy,” “oxidative phosphorylation,” and “mitochondrial inner membrane” (Figure 3C). Intrigued by the prominent enrichment of mitophagy-related pathways, we performed Gene Set Enrichment Analysis (GSEA), which confirmed a significant positive enrichment of gene sets associated with the “regulation of mitophagy” and “mitochondrial degradation involved in mitophagy” in the Icariin-treated group (Figures 3D, E). We thus hypothesized that Icariin enhances metabolic efficiency and reduces lactate accumulation, at least in part, by activating a mitophagic program to clear dysfunctional mitochondria, thereby promoting a healthier mitochondrial pool.

To validate this hypothesis, we first measured the expression of key mitophagy-related genes. Quantitative RT-PCR analysis confirmed that Icariin significantly upregulated the mRNA levels of Pink1, Parkin, Bnip3, and Ulk1 (Figure 3F). To directly visualize and quantify mitophagy, we employed a Mito-Keima assay. Flow cytometric analysis of C2C12 myotubes expressing the pH-sensitive fluorescent probe Mitokeima demonstrated a significant increase in mitophagic flux upon Icariin treatment (Figure 3G). Crucially, this enhanced clearance of damaged organelles was further corroborated by a concomitant decrease in the population of depolarized mitochondria, as quantified by MitoTracker Red CMXRos flow cytometry (Figure 3H). Collectively, these data indicate that Icariin orchestrates a transcriptional program that activates mitophagy, leading to the efficient removal of impaired mitochondria and contributing to the optimization of the mitochondrial network.

### 3.4 4 Icariin activates the master regulator TFEB to drive mitophagic gene expression

**Figure 4:**
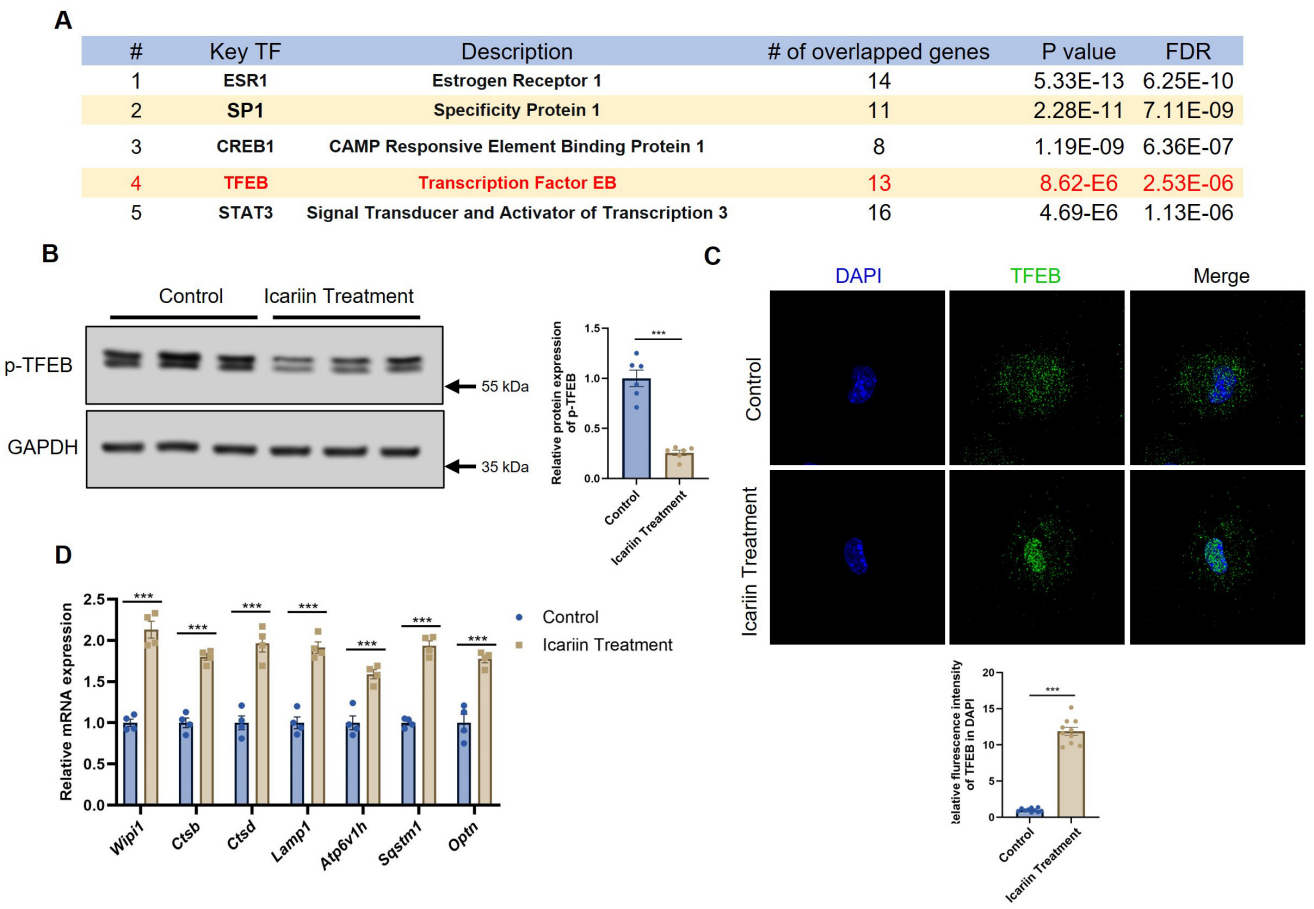
Icariin activates the transcription factor TFEB. **(A)** Prediction of transcription factors potentially regulating the DEGs, analyzed using the TTRUST database. **(B)** Representative west-ern blot and quantification of phosphorylated TFEB (Ser211) and total TFEB protein levels. **(C)** Immunofluorescence staining showing the subcellular localization of TFEB (green) and nuclei (DAPI, blue). Scale bar, 20 μm. **(D)** qRT-PCR analysis of mRNA expression for canonical TFEB downstream target genes. All data are presented as mean ± SEM. Sample size: *n* = 3–4 independent experiments (with 3 technical replicates each), as indicated by the individual data points in the figures. Statistical comparisons between two groups were performed using unpaired two-tailed Student's *t*-test. Significance thresholds: *p < 0.05, **p < 0.01, ***p < 0.001 vs. control group.

To identify the key transcriptional regulators responsible for the observed mitophagic gene signature, we subjected the differentially expressed genes (DEGs) to an analysis using the TTRUST database. This approach pinpointed several top candidate transcription factors, including ESR1, SP1, CREB1, TFEB, and STAT3 (Figure 4A). Among these, TFEB, a master regulator of lysosomal biogenesis and autophagy, emerged as a prime candidate due to its well-established role in coordinating cellular clearance programs.

We therefore investigated whether TFEB is the pivotal mediator of Icariin's effect. TFEB activity is primarily controlled by its subcellular localization; phosphorylation at specific residues (e.g., Ser211) retains it in the cytoplasm in an inactive state, while dephosphorylation promotes its nuclear translocation and transcriptional activation. Western blot analysis revealed that Icariin treatment significantly reduced the level of phosphorylated TFEB (Figure 4B), indicating its activation. Consistent with this dephosphorylation, immunofluorescence staining demonstrated a pronounced translocation of TFEB protein from the cytoplasm into the nucleus following Icariin treatment (Figure 4C). Finally, to confirm the functional outcome of TFEB activation, we examined the expression of its canonical downstream target genes. Quantitative PCR confirmed that Icariin significantly upregulated a panel of TFEB targets involved in lysosomal function and autophagic machinery, including CTSB, CTSD, LAMP1, ATP6V1H, SQSTM1, and OPTN (Figure 4D). Collectively, these results delineate a clear mechanistic pathway whereby Icariin activates TFEB, triggering its nuclear accumulation and the subsequent transactivation of a genetic network essential for mitophagy and lysosomal degradation.

### 3.5 5 TFEB is a master upstream regulator essential for icariin-induced mitophagy and metabolic reprogramming

**Figure 5:**
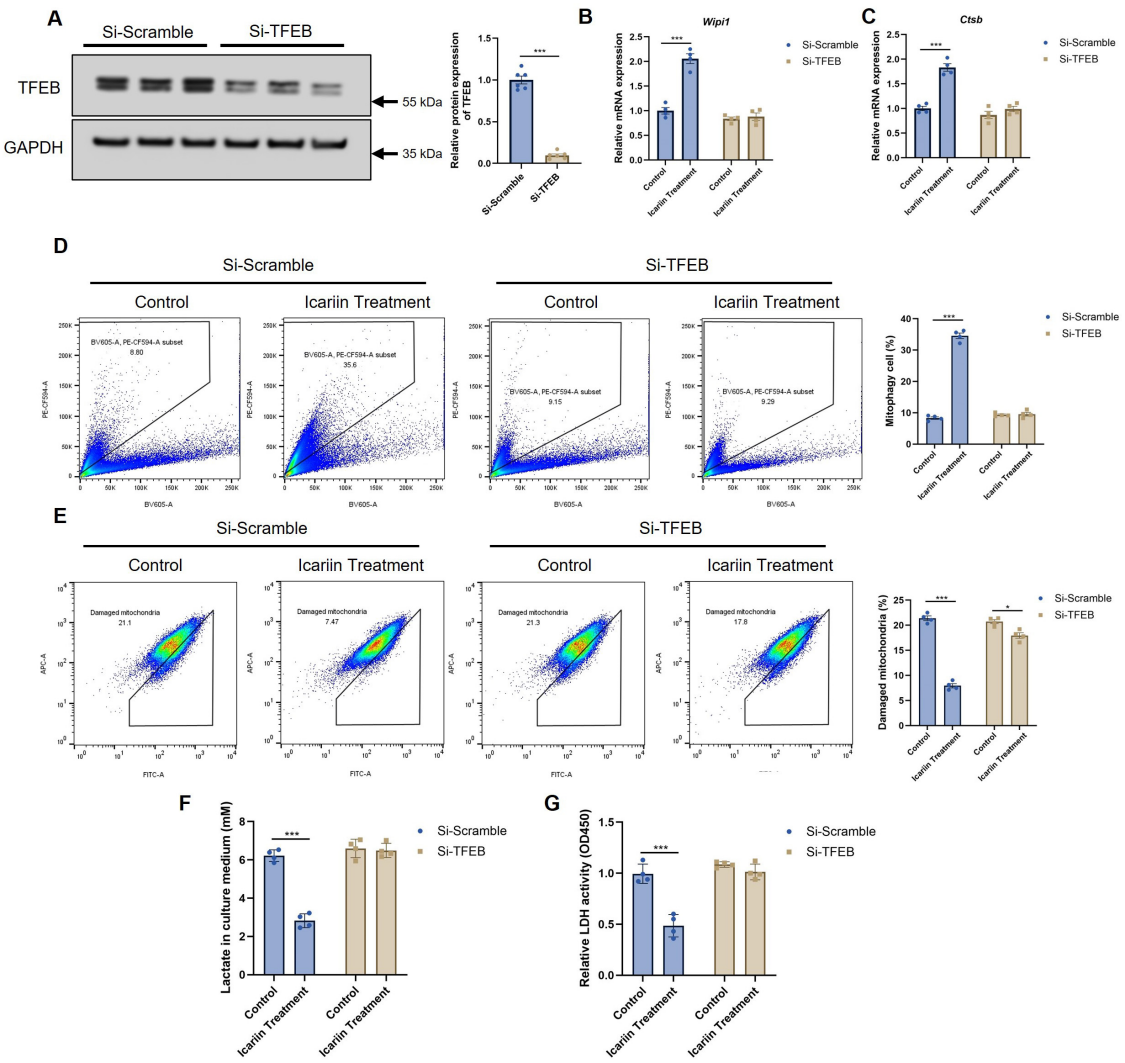
TFEB is required for Icariin-induced effects. **(A)** Western blot analysis confirming TFEB knockdown efficiency in C2C12 myotubes transfected with siRNA. **(B, C)** qRT-PCR analysis of mRNA expression for the mitophagy-related genes WIPI1 **(B)** and CTSB **(C)** in control (si-Scramble) and TFEB-knockdown (si-TFEB) cells treated with or without Icariin. **(D)** Mitophagic flux, measured by Mito-Keima assay, in si-Scramble and si-TFEB cells treated with or without Icariin. **(E)** The proportion of depolarized mitochondria, assessed by MitoTracker Red CMXRos staining, in si-Scramble and si-TFEB cells treated with or without Icariin. **(F)** Lactate concentration in the culture medium of si-Scramble and si-TFEB cells treated with or without Icariin. **(G)** LDH enzyme activity in lysates from si-Scramble and si-TFEB cells treated with or without Icariin. All data are presented as mean ± SEM. Sample size: *n* = 3 independent experiments with 4 technical replicates each, as indicated by the individual data points in the figures. Statistical comparisons between two groups were performed using unpaired two-tailed Student's *t*-test; for multiple groups, one-way ANOVA followed by Tukey's *post hoc* test was used. Significance thresholds: *p < 0.05, **p < 0.01, ***p < 0.001 vs. control group (unless otherwise indicated by brackets).

To establish a definitive causal relationship and ascertain whether TFEB activation sits at the apex of the signaling cascade elicited by Icariin, we conducted a comprehensive loss-of-function study. Genetic ablation of TFEB in C2C12 myotubes via specific siRNA achieved a knockdown efficiency exceeding 90%, effectively crippling this transcriptional master regulator (Figure 5A).

In control (si-Scramble) cells, Icariin treatment potently stimulated the expression of pivotal mitophagy-related genes, including WIPI1 (a phosphatidylinositol-3-phosphate binding protein critical for autophagosome formation) and CTSB (a key lysosomal protease), confirming its role in enhancing the entire autophagic-lysosomal pathway (Figures 5B, C). However, this robust transcriptional response was completely abrogated in TFEB-knockdown (si-TFEB) cells. The failure to induce these genes unequivocally demonstrates that TFEB is the indispensable transcriptional effector required for Icariin to orchestrate the mitophagic gene program.

We next probed the functional ramifications of this transcriptional blockade. Quantitative analysis using the Mito-Keima assay revealed that the significant increase in mitophagic flux instigated by Icariin was entirely nullified in the absence of TFEB (Figure 5D). This finding directly links TFEB-dependent transcription to the execution of functional mitochondrial clearance. Consequently, the Icariin-mediated improvement in mitochondrial health—evidenced by a marked reduction in the population of depolarized mitochondria in control cells—was fully reversed upon TFEB depletion (Figure 5E). The persistence of a damaged mitochondrial pool in si-TFEB cells underscores TFEB's central role in orchestrating the renewal of the mitochondrial network.

Crucially, with the disruption of this mitochondrial quality control axis, the down-stream metabolic benefits of Icariin were entirely eradicated. The characteristic suppression of lactate secretion was no longer observed (Figure 5F). Similarly, the inhibitory effect of Icariin on LDH activity was completely abolished in si-TFEB cells (Figure 5G). This systematic reversal of phenotypes, from gene expression to ultimate metabolic output, provides compelling genetic evidence that TFEB activation is not merely associated with but is fundamentally required for Icariin to enhance mitochondrial function and shift energy metabolism away from glycolysis, thereby elucidating the core mechanism underlying its anti-fatigue efficacy.

## 4 Discussion

Our findings provide compelling evidence that icariin enhances exercise performance through a coordinated multi-level mechanism involving skeletal muscle remodeling, metabolic reprogramming, and enhanced mitochondrial quality control. The results establish a novel signaling pathway where TFEB-mediated mitophagy serves as the central mechanism underlying icariin's anti-fatigue effects.

The observed dose-dependent improvements in aerobic capacity and endurance performance align with previous reports of icariin's anti-fatigue properties (31), but extend our understanding by revealing the precise metabolic basis for these effects. The specific attenuation of exercise-induced lactate accumulation without affecting resting levels suggests that icariin enhances metabolic flexibility during energy demand rather than simply suppressing basal metabolism. This improved metabolic efficiency was accompanied by significant skeletal muscle hypertrophy and a fundamental shift in fiber-type composition toward the oxidative phenotype, consistent with established adaptations to endurance training (32, 33). The enhanced contractile force generation and mitochondrial oxidative capacity provide direct functional validation of these structural and molecular adaptations.

Our *in vitro* investigations revealed crucial insights into icariin's direct cellular actions. The suppression of lactate dehydrogenase activity represents a direct biochemical mechanism limiting lactate production, while the coordinated regulation of monocarboxylate transporters suggests a sophisticated strategy to enhance lactate clearance and utilization (34). Beyond its traditional view as a metabolic byproduct, lactate has emerged as a crucial energy substrate and signaling molecule, serving as a primary carbon source for the tricarboxylic acid (TCA) cycle, particularly during sustained exercise (35, 36). In this context, icariin's ability to modulate lactate metabolism extends beyond merely reducing its accumulation; it likely facilitates the shuttling of lactate toward oxidative tissues, promoting its use as a gluconeogenic precursor and oxidative fuel. This metabolic rerouting not only supports ATP production but also contributes to redox balance and cellular homeostasis (37, 38). This dual approach—reducing production while enhancing clearance—distinguishes icariin from interventions that target only one aspect of lactate metabolism. The metabolic reprogramming toward oxidative phosphorylation provides the fundamental explanation for these effects and is consistent with previous reports of icariin's ability to activate energy-sensing pathways (39, 40).

The transcriptomic analysis revealed a previously unrecognized dimension of icariin's action: the activation of mitophagy. While mitochondrial biogenesis has been well-established in exercise adaptation, the complementary role of mitochondrial quality control through selective autophagy has gained recognition more recently. The enhanced mitophagic flux and concomitant reduction in depolarized mitochondria demonstrate that icariin promotes renewal of the mitochondrial network, ensuring a healthier, more efficient population of organelles (41, 42). This mechanism provides an elegant explanation for the improved mitochondrial function observed both *in vitro* and *in vivo*.

The identification of TFEB as the master regulator of this process represents a significant advancement in understanding how icariin coordinates its pleiotropic effects. TFEB has emerged as a key integrator of cellular clearance programs, and our demonstration of its activation by icariin—through dephosphorylation, nuclear translocation, and transactivation of target genes—places it at the center of the observed metabolic improvements. Most compellingly, the complete abolition of icariin's effects following TFEB knock-down provides definitive genetic evidence for its indispensable role. The systematic reversal of benefits—from gene expression to mitochondrial function to lactate metabolism—establishes a clear causal hierarchy with TFEB at the apex.

This TFEB-mitophagy axis represents a novel mechanism for natural product-mediated enhancement of exercise performance. While other phytochemicals have been shown to activate TFEB, and exercise itself can stimulate mitophagy (43–46), our study is the first to connect these elements into a coherent pathway through which a specific compound enhances fatigue resistance. This finding has broader implications for understanding how nutritional interventions can mimic aspects of exercise adaptation.

Several limitations of our study warrant consideration. First, while we established TFEB's essential role in cultured myotubes, verification in muscle-specific TFEB knockout mice would strengthen the *in vivo* relevance. Second, the precise upstream signaling events leading to TFEB dephosphorylation remain to be elucidated; potential involvement of established regulatory pathways represents an important area for future investigation. Third, the lack of a positive control group in our *in vivo* anti-fatigue experiment is a methodological consideration. While the clear dose-dependent effects observed across multiple functional and molecular parameters provide strong internal validation of icariin's efficacy, the inclusion of a benchmark intervention (e.g., metformin or a known ergogenic aid) would have allowed for direct contextualization of the magnitude of icariin's effects and further verified the responsiveness of the fatigue model. Fourth, our study focused on skeletal muscle, but potential contributions from other tissues to the overall anti-fatigue effects cannot be excluded. Finally, the translation of these findings to human performance requires careful consideration of dosage, timing, and potential individual variability.

Future studies should prioritize the clinical translation of these findings by exploring the therapeutic potential of Icariin in human populations characterized by mitochondrial dysfunction and exercise intolerance, such as the elderly and patients with metabolic diseases. Given that physical exercise—the gold standard intervention—is often limited by adherence or physical capacity in these cohorts, Icariin could serve as a nutritional strategy to mimic or enhance the benefits of endurance training. The temporal dynamics of TFEB activation and mitophagic flux during different exercise regimens also merit investigation, as this could optimize the timing of supplementation to align with endogenous adaptive windows. Furthermore, potential synergistic effects between Icariin and other compounds known to enhance mitochondrial function should be explored for enhanced efficacy, which may ultimately inform the development of combination therapies aimed at improving muscle health and quality of life in aging or diseased states.

In conclusion, our work delineates a complete mechanistic pathway from icariin supplementation to improved exercise performance, with TFEB-mediated activation of mitophagy serving as the central coordinating mechanism. This not only advances our understanding of icariin's anti-fatigue properties but also identifies TFEB as a promising target for interventions aimed at enhancing skeletal muscle metabolic health and exercise capacity.

## Data Availability

The data presented in this study are available from GEO database https://www.ncbi.nlm.nih.gov/geo/ with accession number: GSE313585.
